# Assessment of Medication Adherence and Associated Factors Among Patients With Diabetes Attending a Non-communicable Disease Clinic in a Community Health Centre in Eastern India

**DOI:** 10.7759/cureus.43779

**Published:** 2023-08-20

**Authors:** Abhisek Mishra, Somen K Pradhan, Bimal K Sahoo, Ambarish Das, Arvind K Singh, Swayam Pragyan Parida

**Affiliations:** 1 Community Medicine and Family Medicine, All India Institute of Medical Sciences, Bhubaneswar, Bhubaneswar, IND; 2 Community Medicine, Maharaja Krishna Chandra Gajapati (MKCG) Medical College & Hospital, Berhampur, IND; 3 Community Medicine, Sri Jagannath Medical College & Hospital, Puri, IND; 4 Preventive and Social Medicine, Jawaharlal Institute of Postgraduate Medical Education and Research, Puducherry, IND

**Keywords:** secondary level care hospital, hill-bone medication adherence scale, non-communicable disease, medication adherence, type 2 diabetes patients

## Abstract

Background

Non-adherence to medication represents a modifiable risk factor for patients with type 2 diabetes mellitus (T2DM). Identification of patients with poor adherence can have a significant impact on clinical and socio-economic outcomes in the management of diabetes. This study aimed to assess medication adherence and its associated factors among patients with T2DM attending a non-communicable disease (NCD) clinic in a rural community health centre (CHC) in eastern India.

Methods

The study was a facility-based study that included 207 study participants with an age greater than 18 years. A structured questionnaire was used to collect data on socio-demographic characteristics, health-seeking behaviour, self-care practices, risk factors, clinical information on diabetes, prescription practices, and medication practices. The Hill-Bone Medication Adherence Scale (HB-MAS) has been used to assess medication adherence among study participants.

Results

The study found that the medication adherence rate among the study participants was 67.1%. On multivariate analysis, subjects with social insurance (adjusted odds ratio (AOR) = 2.73, 95% confidence interval (CI) = 1.01-7.38, p-value = 0.047), current smoking status (AOR = 5.47, 95% CI = 1.55-19.23, p-value = 0.008), anxiety (AOR= 3.52, 95% CI= 1.62- 7.61, p-value= 0.001), polypharmacy (AOR= 3.79, 95% CI= 1.25- 11.45, p-value= 0.018), and using alternative medicine (AOR= 5.82, 95% CI= 1.58 - 21.39, p-value= 0.008), were found to have a significantly higher chance of non-adherence. On the other hand, patients practising regular physical activity (AOR = 0.31, 95% CI= 0.12-0.79, p-value = 0.015) and with deprescription (AOR = 0.12, 95% CI= 0.03-0.47, p-value = 0.002) were found to have less chance of non-adherence as compared to their counterparts.

Conclusion

The study highlights the need to identify patients with poor medication adherence and develop interventions according to their requirements through a holistic approach. The study contributes to the existing literature on medication adherence among diabetes patients in rural healthcare settings in eastern India.

## Introduction

Diabetes has reached a significant epidemic proportion in the last few decades. Despite recent improvements in diagnosis and therapeutic management, morbidity and mortality related to type 2 diabetes mellitus (T2DM) continue to increase worldwide [1]. It is projected that diabetes mellitus will affect up to 79.4 million individuals in India by 2030 [2]. On the other hand, the existing rural-urban gap in the burden of diabetes is narrowing rapidly in India [3]. As far as the state of Odisha is concerned, the recently published National Family Health Survey-5 (NFHS-5) of India has reported a higher prevalence of abnormal blood glucose among rural adults in Odisha (men: 15.6%, women: 12.6%) as compared to that at the national level (men: 13.5%, women: 11.4%) [4,5].

Pharmacotherapy, along with lifestyle modifications, remains the mainstay of T2DM management. Medication adherence to pharmacotherapy is key to achieving the intended clinical outcomes during diabetes management. The World Health Organization (WHO) defines adherence as "the extent to which a person’s behaviour- taking medication, following a diet, and/or executing lifestyle changes, corresponds with agreed-upon recommendations from a health care provider" [6]. Despite improving public health care services, medication adherence among patients, particularly those living in rural areas, remains a significant challenge. The recent National Non-Communicable Disease Monitoring Survey (NNMS 2017-18) in India reported that only 26.0% and 4.9% of patients with diabetes from the rural population took prescribed oral medication and insulin, respectively, on a regular basis. At the same time, this survey revealed that only 13.5% of patients with diabetes in rural areas had their blood glucose levels under control [7]. Compliance with recommended medication is crucial to achieving metabolic control, as increased non-adherence to medications among diabetic patients results in a greater likelihood of developing chronic complications and hospitalisation [8]. Several factors, such as low health literacy, lack of access to healthcare providers, financial constraints, and cultural beliefs, have been known to affect medication adherence among patients from rural communities [9].

There is a continuing need to evaluate the level of adherence to medication and emerging factors associated with non-adherence among people with diabetes in local study settings. Despite the wealth of literature available in the field, there is a scarcity of research on patients with diabetes in rural healthcare settings in this part of India. As a result, this study was designed to facilitate the health system in identifying subjects with poor medication adherence, thereby aiding them in planning interventions tailored to the needs of their patients.

The objectives of the present study were to assess medication adherence among patients with T2DM and identify factors associated with the same.

## Materials and methods

Study design and setting

This facility-based observational study was conducted between July and December 2022 at the All India Institute of Medical Sciences, Bhubaneswar, India. This study was performed in the non-communicable disease (NCD) clinic of a Community Health Centre (CHC) situated in one of the coastal blocks of the state of Odisha, India. The NCD clinic offers a broad range of services to patients, which include diagnosis and treatment of NCDs, free medications, and counselling on healthy lifestyles. These patients are managed, treated, and followed up as outpatients or referred to tertiary hospitals as required.

Study population and inclusion/exclusion criteria

Patients with known T2DM, patients staying in the same block for at least one year, and patients who have visited the NCD clinic at least twice within the last six months were included in the study. Patients with comprehension issues due to psychiatric disorders or cognitive impairment were excluded from the study.

Sample size and sampling technique

The sample size was calculated to be 196 using the formula n = z²pq/d² (where z = 1.96, p = prevalence of non-adherence (85%), q = 1 − p, and d (absolute precision) =5%)) [10]. Assuming a 5% non-response rate, a total of 207 patients were recruited for this study as study participants. The study participants were selected using a systematic sampling procedure, in which every third patient meeting the inclusion criteria was included in the study.

Data collection

Data collection was done by administering a structured questionnaire that contained details such as socio-demographic characteristics, health-seeking behaviour, self-care practices, risk factors, clinical information on diabetes, prescription practices, and medication practices. The questionnaire was pretested on 20 subjects (10% of the total sample size), and necessary modifications were incorporated before administration. Adherence was assessed using the nine-item Hill-Bone Medication Adherence Scale (HB-MAS), which has already been used by researchers in the least developed and developing countries, including India (Appendix A). This is a nine-item questionnaire measuring adherence on a 4-point Likert scale. The raw score was converted to a percentage by dividing the actual score by the total possible score and multiplying the result by 100. In the study, non-adherence was defined as an HB-MAS score of less than 80%. [11-13] Similarly, the Generalised Anxiety Disorder Assessment (GAD-7) and Patient Health Questionnaire (PHQ-9) were used to assess anxiety and depression, respectively, among the study participants (Appendices B-C). [14,15] The linguistic validation process was undertaken to ensure that an accurately translated Odia version of the questionnaire is used in our study. This process involved all the steps, i.e., forward translation, harmonisation, back translation, cognitive debriefing, review, and finalisation, to guarantee that the translated version is a faithful representation of the original version. [16] For this study, polypharmacy was defined as the number of drugs consumed by the patient being more than or equal to five. Similarly, deprescription was defined as tapering, stopping, discontinuing, or withdrawing any single or multiple drugs previously consumed by the patient in the last visit. The respective attending physician completed the questionnaire by interviewing the participant and reviewing the patient's medical records. Simultaneously, data were entered using Epicollect5 software (Centre for Genomic Pathogen Surveillance, 2023, v4.2.0).

Data analysis

Descriptive analyses were performed to evaluate the sociodemographic, clinical, and pharmacological characteristics of the study population. Univariate and multivariate binary logistic regression analyses were performed for non-adherence using a range of variables as independent predictors. For all statistical tests, an alpha level of 0.05 was used to detect statistical significance. All statistical analyses were executed using IBM SPSS Statistics software version 26 (IBM Corp., Armonk, NY, USA).

Ethical considerations

Ethical approval was obtained from the Institutional Ethics Committee of All India Institute of Medical Sciences, Bhubaneswar (T/IM-NF/CM&FM/21/52). Informed written consent was obtained from the participants for the interviews, and they could opt-out at any time during the study.

## Results

In total, 207 patients were recruited during the study period. The mean ± SD age of the patients was 54.29 + 9.51 years, and the majority (59.90%) were female. The maximum proportion (64.3%) of participants had a history of more than five visits to the NCD clinic during the last six months. On assessment, 78 (37.69%) participants were found to be nonadherent to anti-diabetic medications. The basic characteristics of the participants are listed in Table 1.

**Table 1:** Basic characteristics of study participants NCD: non-communicable disease

Sl.No.	Characteristics		N (%)
1	Gender	Male	83 (40.10%)
		Female	124 (59.90%)
2	Highest level of education	Illiterate	61 (29.50%)
		Primary	27 (13.00%)
		Middle	75(36.20%)
		Secondary	22 (10.60%)
		Intermediate	11 (5.30%)
		Graduate and above	11 (5.30%)
3	Number of prior visits to the NCD clinic	Two visits	37 (17.9%)
		Three to five visits	37 (17.9%)
		More than five visits	133 (64.3%)
4	Medication adherence	Adherent	129 (62.31%)
		Non-adherent	78 (37.69%)

Univariate binary logistic regression tests were performed against various independent variables to determine predictors of medication adherence, as shown in Tables 2-6.

**Table 2:** Univariate analysis of socio-demographic factors with medication adherence UOR: unadjusted odds ratios; OBC: Other Backward Classes; SC: Scheduled Castes; ST: Scheduled Tribes; APL: Above Poverty Line; BPL: Below Poverty Line

Sl.No.	Factors		Medication adherence				UOR	p-value
			Adherent		Non-adherent			
			N	%	N	%		
1	Age group	<50 years	43	33.33%	28	35.90%	Reference	
		>50 years	86	66.67%	50	64.10%	0.89 (0.50-1.61)	0.707
2	Gender	Female	75	58.14%	49	62.82%	Reference	
		Male	54	41.86%	29	37.18%	0.82 (0.46-1.46)	0.506
3	Education	Illiterate	38	29.46%	23	29.49%	Reference	
		Literate	91	70.54%	55	70.51%	1.00 (0.54-1.85)	0.996
4	Marital status	Married	111	86.05%	62	79.49%	Reference	
		Unmarried	1	0.78%	3	3.85%	5.37 (0.55-52.74)	0.149
		Widow/Separated	17	13.18%	13	16.67%	1.37 (0.62-3.01)	0.434
5	Family structure	Joint/Extended	82	63.57%	33	42.31%		
		Nuclear	47	36.43%	45	57.69%	2.38 (1.34-4.23)	0.003
6	Occupation	Employed	48	37.21%	25	32.05%	Reference	
		Retired	7	5.43%	6	7.69%	1.65 (0.50-5.42)	0.413
		Unemployed	74	57.36%	47	60.26%	1.22 (0.67-2.24)	0.521
7	Social class	General	66	51.16%	28	35.90%	Reference	
		OBC	45	34.88%	32	41.03%	1.68 (0.89-3.16)	0.110
		SC/ST	18	13.95%	18	23.08%	2.36 (1.07-5.19)	0.033
8	APL/BPL	APL	45	34.88%	21	26.92%	Reference	
		BPL	84	65.12%	57	73.08%	1.45 (0.78-2.70)	0.235
9	Social Insurance	No	40	31.01%	11	14.10%	Reference	
		Yes	89	68.99%	67	85.90%	2.74 (1.31-5.73)	0.008
10	Health insurance	No	56	43.41%	29	37.18%	Reference	
		Yes	73	56.59%	49	62.82%	1.30 (0.73-2.31)	0.378

**Table 3:** Univariate analysis of health-seeking behaviour and self-care practices with medication adherence UOR: unadjusted odds ratios; DM: diabetes mellitus; BP: blood pressure

Sl.No.	Factors		Medication adherence				UOR	p-value
			Adherent		Non-adherent			
			N	%	N	%		
1	Visiting doctor	Both public and private hospitals	12	9.30%	20	25.64%	Reference	
		Public hospital	117	90.70%	58	74.36%	0.30 (0.14-0.65)	0.002
2	Family history of DM	No	89	68.99%	56	71.79%	Reference	
		Yes	40	31.01%	22	28.21%	0.87 (0.47-1.62)	0.670
3	Self-monitoring BP	No	124	96.12%	77	98.72%	Reference	
		Yes	5	3.88%	1	1.28%	0.32 (0.04-2.81)	0.305
4	Self-monitoring of blood glucose	No	116	89.92%	71	91.03%	Reference	
		Yes	13	10.08%	7	8.97%	0.88 (0.34-2.31)	0.795
5	Self-monitoring of foot	No	109	84.50%	70	89.74%	Reference	
		Yes	20	15.50%	8	10.26%	0.62 (0.26-1.49)	0.288

**Table 4:** Univariate analysis of risk factors with medication adherence UOR: unadjusted odds ratios; T2DM: type 2 diabetes mellitus

Sl.No.	Factors		Medication adherence				UOR	p-value
			Adherent		Non-adherent			
			N	%	N	%		
1	Smoking status	Non-user	7	5.43%	5	6.41%	Reference	
		Current user	8	6.20%	9	11.54%	1.58(0.35-7.00)	0.551
		Ex-user	114	88.37%	64	82.05%	0.79(0.24-2.58)	0.691
2	Smokeless tobacco use	Non-user	44	34.11%	22	28.21%	Reference	
		Current user	9	6.98%	14	17.95%	3.11 (1.17-8.30)	0.023
		Ex-user	76	58.91%	42	53.85%	1.11 (0.59-2.09)	0.758
3	Alcohol	Non-user	7	5.43%	2	2.56%	Reference	
		Current user	7	5.43%	9	11.54%	4.5 (0.70-28.79)	0.112
		Ex-user	115	89.15%	67	85.90%	2.04 (0.41-10.10)	0.383
4	Physical activity	No	83	64.34%	69	88.46%	Reference	
		Yes	46	35.66%	9	11.54%	0.24 (0.11-0.52)	0.001<
5	Consumes recommended diet	No	65	50.39%	57	73.08%	Reference	
		Yes	64	49.61%	21	26.92%	0.37 (0.20-0.69)	0.002
6	Anxiety	No	93	72.09%	35	44.87%	Reference	
		Yes	36	27.91%	43	55.13%	3.17 (1.76-5.72)	0.001<
7	Depression	No	94	72.87%	55	70.51%	Reference	
		Yes	35	27.13%	23	29.49%	1.12 (0.60-2.09)	0.715
8	Any comorbidity	No	65	50.39%	38	48.72%	Reference	
		Yes	64	49.61%	40	51.28%	1.07 (0.61-1.88)	0.816
9	Family history of T2DM	No	89	68.99%	56	71.79%	Reference	
		Yes	40	31.01%	22	28.21%	0.87 (0.47-1.62)	0.670

**Table 5:** Univariate analysis of complications with medication adherence UOR: unadjusted odds ratios

Sl.No.	Factors		Medication adherence				UOR	p-value
			Adherent		Non-adherent			
			N	%	N	%		
1	Retinopathy	Absent	10	7.75%	9	11.54%	Reference	
		Present	2	1.55%	4	5.13%	2.22 (0.33-15.18)	0.415
		Unknown status	117	90.70%	65	83.33%	0.61 (0.24-1.60)	0.320
2	Neuropathy	Absent	61	47.29%	23	29.49%	Reference	
		Present	68	52.71%	55	70.51%	2.15 (1.18-3.90)	0.012
3	Nephropathy	Absent	7	5.43%	10	12.82%	Reference	
		Present	1	0.78%	3	3.85%	2.1 (0.18-24.60)	0.555
		Unknown status	121	93.80%	65	83.33%	0.38 (0.14-1.03)	0.058
4	Foot ulcer	Absent	127	98.45%	73	93.59%	Reference	
		Present	2	1.55%	5	6.41%	4.35 (0.82-22.99)	0.084

**Table 6:** Univariate analysis of prescription and medication practices with medication adherence ^†^ Include natural or traditional healing methods, such as Ayurveda, Yoga and Naturopathy, Unani, Siddha, and Homeopathy UOR: unadjusted odds ratios; Govt.: government

Sl.No.	Factors		Medication adherence				UOR	p-value
			Adherent		Non-adherent			
			N	%	N	%		
1	Polypharmacy	No	121	93.80%	55	70.51%	Reference	
		Yes	8	6.20%	23	29.49%	6.32 (2.66-15.03)	0.001<
2	Deprescription	No	102	79.07%	73	93.59%	Reference	
		Yes	27	20.93%	5	6.41%	0.259 (0.10-0.70)	0.008
3	Using insulin	No	105	81.40%	73	93.59%	Reference	
		Yes	24	18.60%	5	6.41%	0.3 (0.11-0.82)	0.019
4	Use of alternative medicine ^†^	No	122	94.57%	54	69.23%	Reference	
		Yes	7	5.43%	24	30.77%	7.75 (3.14-19.07)	0.001<
5	Drug bought from	Both	27	20.93%	16	20.51%	Reference	
		Govt. pharmacy	95	73.64%	57	73.08%	1.01 (0.50-2.04)	0.972
		Private pharmacy	7	5.43%	5	6.41%	1.21 (0.33-4.44)	0.779
6	Remembers medicine intake	Others	3	2.33%	5	6.41%	Reference	
		Self	87	67.44%	36	46.15%	0.25 (0.06-1.09)	0.066
		Both	39	30.23%	37	47.44%	0.57 (0.13-2.55)	0.462
7	Brings medicine	Others	2	1.55%	3	3.85%	Reference	
		Self	87	67.44%	38	48.72%	0.29 (0.05-1.81)	0.186
		Both	40	31.01%	37	47.44%	0.62 (0.10-3.90)	0.607

Similarly, Table 7 demonstrates the multivariate regression model, showing the significant factors involved in predicting medication adherence among T2DM patients.

**Table 7:** Multivariate logistic regression analysis of predictors with medication adherence AOR: adjusted odds ratio; OBC: Other Backward Classes; SC: Scheduled Castes; ST: Scheduled Tribes

Sl.No.	Factors		Medication adherence				AOR	p-value
			Adherent		Non-adherent			
			N	%	N	%		
1	Family structure	Joint/Extended	82	63.57%	33	42.31%	Reference	
		Nuclear	47	36.43%	45	57.69%	1.24 (0.55-2.8)	0.605
2	Social class	General	66	51.16%	28	35.90%	Reference	
		OBC	45	34.88%	32	41.03%	1.62 (0.72-3.63)	0.244
		SC/ST	18	13.95%	18	23.08%	1.66 (0.56-4.95)	0.363
3	Social insurance	No	40	31.01%	11	14.10%	Reference	
		Yes	89	68.99%	67	85.90%	2.73 (1.01-7.39)	0.047
4	Visiting doctor	Both public and private hospitals	12	9.30%	20	25.64%	Reference	
		Public hospital	117	90.70%	58	74.36%	0.73 (0.23-2.34)	0.599
5	Smokeless tobacco use	Non-user	44	34.11%	22	28.21%	Reference	
		Current user	9	6.98%	14	17.95%	5.47 (1.56-19.24)	0.008
		Ex-user	76	58.91%	42	53.85%	1.53 (0.68-3.51)	0.305
6	Physical activity	No	83	64.34%	69	88.46%	Reference	
		Yes	46	35.66%	9	11.54%	0.31 (0.12-0.80)	0.015
7	Consumes recommended diet	No	65	50.39%	57	73.08%	Reference	
		Yes	64	49.61%	21	26.92%	0.85 (0.38-1.90)	0.699
8	Anxiety	No	93	72.09%	35	44.87%	Reference	
		Yes	36	27.91%	43	55.13%	3.52 (1.63-7.61)	0.001
9	Neuropathy	Absent	61	47.29%	23	29.49%	Reference	
		Present	68	52.71%	55	70.51%	1.86 (0.857-4.03)	0.117
10	Polypharmacy	No	121	93.80%	55	70.51%	Reference	
		Yes	8	6.20%	23	29.49%	3.80 (1.26-11.45)	0.018
11	Deprescription	No	102	79.07%	73	93.59%	Reference	
		Yes	27	20.93%	5	6.41%	0.13 (0.03-0.48)	0.002
12	Using insulin	No	105	81.40%	73	93.59%	Reference	
		Yes	24	18.60%	5	6.41%	0.43 (0.13-1.46)	0.177
13	Use of alternative medicine	No	122	94.57%	54	69.23%	Reference	
		Yes	7	5.43%	24	30.77%	5.82 (1.59-21.39)	0.008

Among socio-demographic factors, univariate logistic regression analysis revealed that subjects with nuclear families belonging to Scheduled Castes (SC) and Scheduled Tribes (ST) and those with social insurance had a higher risk of non-adherence than their counterparts (Table 2).

Regarding health-seeking behaviour, univariate analysis showed that participants visiting only public hospitals (UOR = 0.297, 95% CI= 0.136-0.650, p-value = 0.002) had a lower chance of non-adherence than those visiting public and private hospitals (Table 3).

On univariate analysis of various risk factors, the absence of adequate physical activity, non-consumption of the recommended diet, and anxiety had a poor effect on medication adherence (Table 4).

Similarly, on univariate analysis of various disease-related complications, none of the factors had a predictive effect on medication adherence (Table 5).

Univariate analysis of prescription and medication practices with medication adherence reported polypharmacy, deprescription, using insulin, and use of alternative medicine as significant factors having a negative impact on medication adherence (Table 6).

On multivariate analysis, while adjusting for other factors, subjects with social insurance (AOR = 2.733, 95% CI= 1.011-7.385, p-value = 0.047) had a significantly higher chance of non-adherence. On the assessment of various risk factors, subjects with current smoking status (AOR = 5.470, 95% CI= 1.555-19.239, p-value = 0.008) and anxiety (AOR = 3.520, 95% CI= 1.629-7.610, p-value = 0.001) were found to have a higher risk of non-adherence. Similarly, those practising regular physical activity (AOR = 0.311, 95% CI= 0.121-0.799, p-value = 0.015) had a lesser chance of becoming non-adherent to medication. Patients with polypharmacy (AOR = 3.797, 95% CI= 1.259-11.453, p-value = 0.018) and those using alternative medicine (AOR = 5.827, 95% CI= 1.587-21.390, p-value = 0.008) were likely to become more non-adherent. On the other hand, participants with deprescription (AOR = 0.127, 95% CI= 0.034-0.475, p-value = 0.002) were found to have a lesser chance of non-adherence than their counterparts (Table 7).

## Discussion

In this study, approximately two-fifths (37.69%) of the participants were non-adherent to anti-diabetic medications. The estimated non-adherence in the present study was found to be much lower as compared to that of similar studies done in Odisha by Swain et al. (85%) and Sahoo et al. (65.66%) [10,17]. Both of these studies were conducted among urban residents with T2DM visiting government and private tertiary care and teaching hospitals, respectively. The presence of free drug distribution schemes by the government in public health facilities may explain the better adherence among our study participants compared to that of the study by Sahoo et al.. Similarly, a separate biweekly NCD clinic in a primary healthcare setting may also have added to the medication adherence practice among the current study participants due to better accessibility than the other two studies [18]. Interestingly, our study reported a negative impact of the presence of social insurance on the medication adherence of participants. Individuals with social health insurance may be less interested in preventive health interventions due to various factors, such as personal beliefs, a lack of trust in the healthcare system, or the belief that they are not exposed to financial risk for disease complications. Hernandez et al. reported no significant effect of social insurance on various healthcare process indicators, including self-care practices, among patients with diabetes [19]. A significant association of medication adherence with several socio-demographic factors can be explained by the influence of these factors on health-seeking behaviour among participants [20].

Our study reported an increased risk of non-adherence among participants with current tobacco use and inadequate physical activity. This may be because the clustering of unhealthy behaviours is an observed phenomenon, and it would be expected that patients exposed to unhealthy lifestyles would also show poor adherence to their prescribed medication [21]. The present study also found a significant association between anxiety and medication adherence. A similar study conducted by James E. Aikens reported the presence of diabetes-related distress (DRD), depression, and anxiety as prognostic indicators of the impending decline in numerous aspects of diabetes self-management, including medication adherence [22].

Prescription-related factors like deprescription and polypharmacy were among the significant predictors of medication adherence, having a positive and negative impact on patient adherence to prescribed medicines, respectively. This is in contrast to a similar study conducted by Takeshi et al. in Japan, who reported that polypharmacy was associated with improved adherence [23]. In this study, subjects using alternative medicine showed a higher chance of non-adherence than those not using the same. This finding is consistent with that of a study by Sharma et al. in India [24]. These differences can be due to fear of unreported side effects, a lack of confidence in immediate or future benefits, and variation in health literacy leading to suboptimal medication adherence [8].

Our results should be viewed with consideration of several limitations. One primary limitation was that we included only those patients who visited the clinic, and those who did not visit were excluded from the study. Similarly, as we have collected self-reported data on medication adherence, there is a possible tendency to overestimate adherence due to recall biases and social desirability. Despite these limitations, this study provides valuable information that supports the literature and has several strengths. This is one of the few studies conducted at the rural healthcare facility level in this geographical area. In addition, this is among the selected studies that try to explore the effect of mental health factors on medication adherence among patients with diabetes.

## Conclusions

The purpose of this study was to determine the various factors affecting medication adherence among people with diabetes in a rural healthcare setting. These findings suggest that, in general, non-adherence remains a challenge for the health system despite improvements in healthcare service delivery at the grassroots level in India. This study showed that lifestyle behaviour, prescription practices, and mental health factors are crucial in determining medication adherence among T2DM patients. Therefore, there is a definite need for an individualised approach towards diabetes patients during counselling and treatment, with particular emphasis on shared decision-making between providers and patients. Some of the key policy priorities should be the inclusion of a fixed-dose combination of anti-diabetic drugs and strengthening patient education programmes in the public healthcare delivery system. Making medication adherence assessments part of routine NCD care can go a long way in bringing the health system a step closer to achieving improved clinical outcomes among T2DM patients.
